# Extensor Mechanism Reconstruction Following Periprosthetic Joint Infection After Total Knee Arthroplasty: A Systematic Review

**DOI:** 10.7759/cureus.116208

**Published:** 2026-09-14

**Authors:** Camilla Prosenz, Hisham Fansa, Hannah Engel, Aikaterini Ioannou, Alexander Eberle, Markos Ioannou

**Affiliations:** 1 Orthopaedics and Traumatology, Spital Zollikerberg, Zurich, CHE; 2 Plastic and Reconstructive Surgery, Spital Zollikerberg, Zurich, CHE; 3 Medicine and Surgery, University of Patras, Patras, GRC; 4 Ophthalmology, Vista Klinik, Basel, CHE; 5 Orthopaedics, Spital Zollikerberg, Zurich, CHE

**Keywords:** allograft, extensor mechanism reconstruction, knee society score, periprosthetic joint infection, synthetic mesh, systematic review, total knee arthroplasty

## Abstract

Periprosthetic joint infection (PJI) with concomitant extensor mechanism disruption (EMD) following total knee arthroplasty (TKA) is a rare but devastating complication associated with severe functional loss and limited limb-salvage options. Although several reconstructive strategies have been described, outcomes in the infected setting remain poorly defined. We conducted a systematic review, in accordance with the Preferred Reporting Items for Systematic Reviews and Meta-Analyses (PRISMA) 2020 guidelines, to evaluate the reconstructive approaches and reported clinical outcomes for EMD in the setting of PJI after TKA. PubMed and Semantic Scholar were searched from inception through January 31, 2026, for studies reporting outcomes after extensor mechanism reconstruction in patients with PJI following TKA; primary clinical studies and systematic reviews were eligible. Extracted variables included reconstructive technique, failure and reinfection rates, functional outcomes, and follow-up duration. Eleven studies met the inclusion criteria, comprising three primary clinical studies and eight systematic reviews or meta-analyses. Reported failure rates varied across the included literature, and no formal comparison established superiority or equivalence of allograft versus synthetic mesh reconstruction. Reconstruction was associated with meaningful functional improvement: postoperative Knee Society Scores frequently exceeded 70 points, extensor lag typically improved to less than 10°, and postoperative flexion generally ranged from 95° to 110°. Recurrent infection remained an important cause of treatment failure in the PJI-specific literature. Extensor mechanism reconstruction after PJI complicating TKA remains a complex limb-salvage procedure with a guarded prognosis; reported outcomes appear to depend on infection control, soft-tissue quality, and defect-specific reconstructive planning.

## Introduction and background

Total knee arthroplasty (TKA) is among the most commonly performed orthopedic procedures worldwide, and although outcomes are generally excellent, serious complications can occur, one of the most consequential being periprosthetic joint infection (PJI) [1]. PJI occurs in approximately 0.5-2% of primary TKA cases and increases to 2-4% following revision surgery [2].

PJI is a leading cause of revision TKA failure, and its precipitating factors are multifactorial [2]. A detailed analysis of these factors is beyond the scope of the present review, which focuses on reconstructive management after PJI-associated extensor mechanism disruption (EMD) has occurred.

EMD is a separate, equally devastating complication that results in loss of active knee extension, with reported incidence rates ranging from 0.1% to 2.5% [3-5]. It may arise from rupture of the quadriceps tendon, patellar fracture, or disruption of the patellar tendon [6]. Despite its low incidence, EMD poses a major reconstructive challenge, particularly in the post-arthroplasty setting [6].

The extensor mechanism of the knee is a continuous musculotendinous chain comprising the quadriceps tendon, the patella, and the patellar tendon, which together transmit the force generated by the quadriceps muscle to the tibial tuberosity, enabling active knee extension. Disruption at any point along this chain, whether at the quadriceps tendon, the patella itself, or the patellar tendon, results in loss of active extension and is therefore functionally equivalent in its clinical consequences, even though the anatomic level of injury differs.

Primary repair of the extensor mechanism, although reliable in native knees, yields considerably worse outcomes after TKA, with high re-rupture rates reported following direct repair in the periprosthetic setting [6,7]. These results reflect the altered biomechanical environment and impaired local healing potential of the post-arthroplasty knee, including altered local vascularity, peri-implant scarring, and impaired tendon quality. Consequently, reconstruction using allografts, autografts, or synthetic mesh has emerged as the preferred surgical approach in these patients [5,7].

The simultaneous presence of PJI further complicates extensor mechanism reconstruction [2]. Infection compromises local tissue biology and restricts the safe use of foreign material, making durable reconstruction particularly challenging [2]. Contemporary strategies therefore aim to restore extensor continuity while achieving lasting infection control, an objective that remains difficult to accomplish in clinical practice [2].

When complex soft-tissue defects accompany the extensor mechanism deficiency, additional coverage with pedicled or free flaps is frequently required [8]. Historically, the pedicled gastrocnemius muscle flap has been the standard option for soft-tissue reconstruction around the knee [8]. Advances in reconstructive microsurgery have expanded the armamentarium to include anterolateral thigh and perforator-based free flaps; however, comparative data on functional outcomes, limb salvage, morbidity, and quality of life across these techniques remain limited [7,8].

A further challenge is that much of the existing literature does not clearly distinguish between EMD arising from trauma and that occurring in the context of PJI, despite the markedly different biological and mechanical demands these scenarios present. Evidence specifically addressing extensor mechanism reconstruction in the setting of PJI therefore remains limited and fragmented, consisting mainly of small retrospective case series and heterogeneous pooled cohorts.

The present systematic review therefore aimed to synthesize the available evidence on reconstructive strategies and reported outcomes for EMD following PJI after TKA.

## Review

Materials and methods

This systematic review was conducted and is reported in accordance with the Preferred Reporting Items for Systematic Reviews and Meta-Analyses (PRISMA) 2020 guidelines [9]. The review was not prospectively registered in the International Prospective Register of Systematic Reviews (PROSPERO); it was undertaken as a targeted synthesis of an underreported clinical entity for which no registered protocol existed. The absence of prospective registration is acknowledged as a methodological limitation.

Literature Search

A systematic search of PubMed and Semantic Scholar was performed from database inception through January 31, 2026. The search combined controlled-vocabulary (Medical Subject Headings (MeSH)) terms with free-text keywords. The following string was used for PubMed: ("extensor mechanism"[Title/Abstract] OR "extensor apparatus"[Title/Abstract]) AND ("reconstruction"[Title/Abstract] OR "repair"[Title/Abstract]) AND ("total knee arthroplasty"[Title/Abstract] OR "total knee replacement"[Title/Abstract] OR TKA[Title/Abstract]) AND ("infection"[Title/Abstract] OR "periprosthetic joint infection"[Title/Abstract] OR PJI[Title/Abstract]).

The following MeSH terms were additionally applied: ("Knee Arthroplasty"[MeSH]) AND ("Surgical Wound Infection"[MeSH] OR "Prosthesis-Related Infections"[MeSH]). The search strategy was adapted accordingly for Semantic Scholar: the same core keyword combinations used for PubMed were applied as free-text terms, without field-specific tags or MeSH mapping, as the platform does not support these functions. No language, publication-date, or study-design restrictions were applied. Reference lists of all included articles were screened manually for additional eligible publications. Embase, Web of Science, and the Cochrane Library were not searched. This restricted database coverage is acknowledged as a limitation.

Eligibility Criteria

Primary clinical studies were eligible if they enrolled patients who had undergone TKA and subsequently developed PJI, reported at least one clinical or functional outcome after surgical reconstruction of the extensor mechanism (using allograft, synthetic mesh, autograft, or a combination), and included a minimum of two patients. Systematic reviews addressing extensor mechanism reconstruction after TKA were also eligible as contextual secondary evidence, including reviews with mixed infected and non-infected cohorts. Single-patient case reports and conference abstracts without an eligible cohort were excluded. Eligible primary-study designs included case series, cohort studies, case-control studies, and randomized controlled trials. Studies reporting outcomes exclusively in native-knee or non-arthroplasty populations were excluded.

Study Selection and Data Extraction

Records were screened by one reviewer (C.P.) on the basis of title and abstract; potentially eligible studies were then assessed in full text, and final inclusion was decided together with a second author (M.I.) by consensus. Eleven studies were included from an initial pool of 318 records identified across both databases. This screening and consensus process did not constitute independent dual-reviewer screening.

Data were extracted by one reviewer (C.P.) according to a predefined list of data items and reviewed together with a second author (M.I.), with any discrepancies resolved by discussion. Extracted variables included patient demographics and number of reconstructed knees; presence of active infection at reconstruction; history of prior failed reconstruction; anatomic location and chronicity of EMD; graft material, surgical technique, and augmentation strategies; definition and rate of reconstruction failure; functional outcome scores (Knee Society Score (KSS) [10]); postoperative range of motion; extensor lag; walking-aid requirement; complication rates (reinfection, re-rupture, wound complications); and duration of follow-up. This process did not constitute independent dual data extraction.

Risk of Bias and Level of Evidence

Methodological quality was appraised with design-appropriate tools: the Joanna Briggs Institute (JBI) critical appraisal checklist for case series [11] for the three primary studies and the ROBIS (Risk of Bias in Systematic Reviews) tool [12] for the eight included systematic reviews. The appraisal was carried out by one reviewer (C.P.) and reviewed together with a second author (M.I.), with the final ratings agreed by consensus. The body of evidence corresponds to Level IV, reflecting its basis in Level IV observational studies and secondary syntheses thereof.

No de novo meta-analysis was undertaken. This reflects the absence of a sufficiently common and clinically interpretable estimand across the available PJI-specific studies, together with non-independence among the secondary reviews, whose included populations, reconstruction techniques, definitions of failure, units of analysis, and follow-up durations differed materially. A random-effects model can accommodate some between-study heterogeneity, but it does not make fundamentally different outcome definitions comparable and does not resolve non-independence created by the repeated inclusion of the same primary studies across multiple reviews. Outcomes were therefore synthesized narratively, with the PJI-specific primary studies treated as the core evidence and the broader systematic reviews used as contextual secondary evidence; estimates from these two evidence levels are reported separately and are not combined into a new pooled or weighted summary.

Results

Literature Search

The database search and screening process is summarized in Figure 1. Of 318 records identified, 11 studies satisfied the inclusion criteria and were included in the qualitative synthesis [3,4,6,13-20]. The seven reports excluded at full-text assessment did not specifically address extensor mechanism outcomes in the setting of infection.

**Figure 1:**
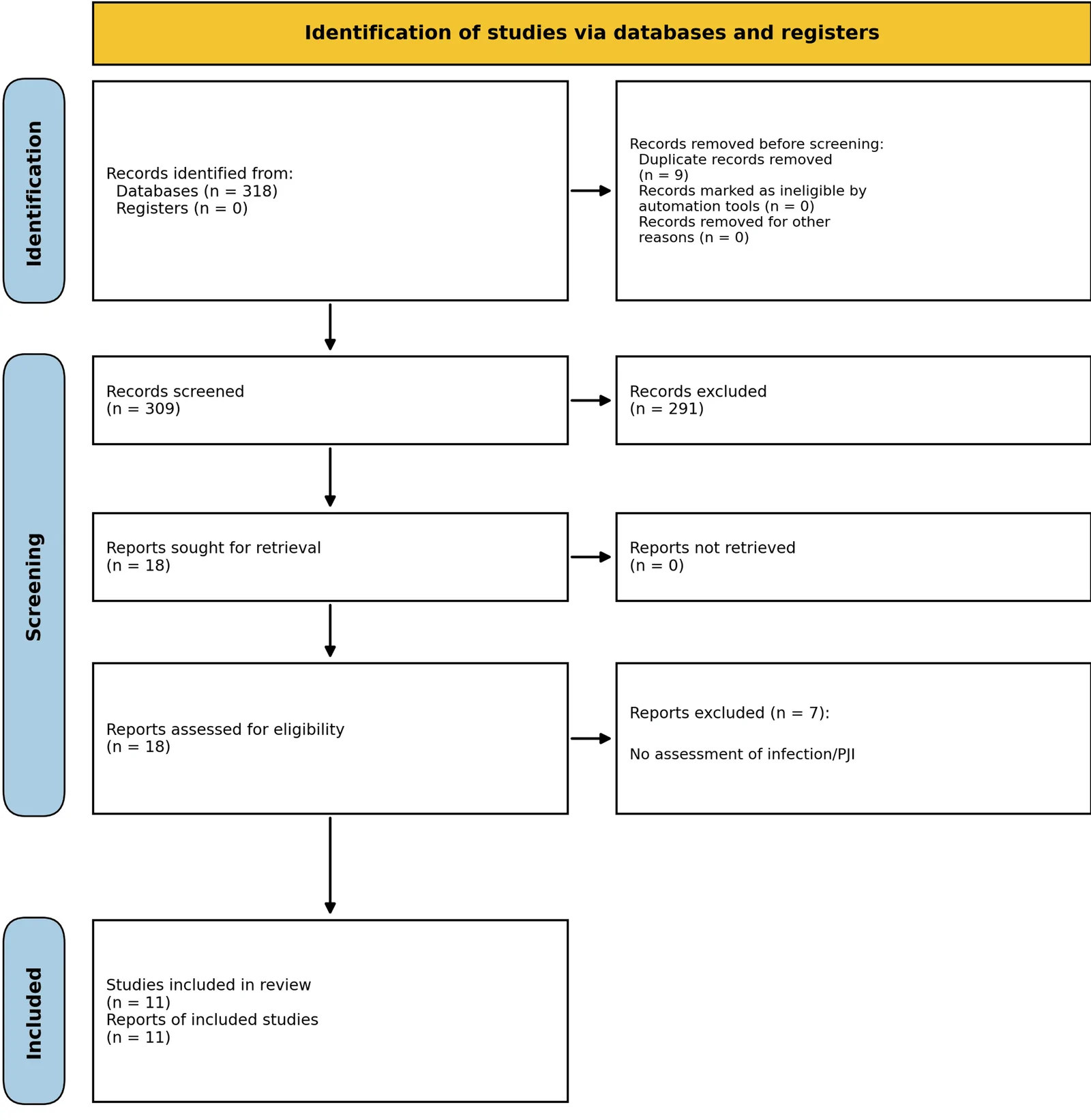
PRISMA 2020 flow diagram of the literature search and study selection Records were identified through PubMed and Semantic Scholar; after the removal of duplicates and the screening of titles and abstracts, full-text assessment yielded 11 included studies. PRISMA: Preferred Reporting Items for Systematic Reviews and Meta-Analyses; PJI: periprosthetic joint infection

Characteristics of the Included Studies

The 11 included sources comprised three primary clinical studies and eight systematic reviews or meta-analyses that variably included infected and non-infected TKA cohorts (Table 1). Two of the primary studies (Perry et al. [13] and Kyriakidis et al. [14]) addressed PJI-associated EMD exclusively, whereas the orthoplastic series of Osinga et al. [17] reported a broader PJI cohort in which extensor mechanism deficiency was a subset. Because dedicated primary evidence on this combined pathology is sparse, the three primary studies were treated as the core evidence base, and the previously published systematic reviews were used chiefly to provide supporting context on reconstructive strategies and pooled outcomes rather than as independent primary data.

**Table 1:** Overview of the included studies reporting extensor mechanism reconstruction after TKA, including study design, patient characteristics, reconstructive techniques, failure rates, and follow-up ALT: anterolateral thigh; ATA: Achilles tendon allograft; EMA: extensor mechanism allograft; EMD: extensor mechanism disruption; MSAP: medial sural artery perforator; PJI: periprosthetic joint infection; TFL: tensor fascia lata; TKA: total knee arthroplasty

Study	Study type	Study title	Sample size	Reconstruction technique	Failure rate and key results	Mean follow-up
Perry et al., 2018 [13]	Retrospective case series; single center	Two-stage exchange and Marlex-mesh reconstruction for infection with extensor mechanism disruption after total knee arthroplasty	16 knees; all TKA, EMD, PJI	Synthetic mesh (Marlex)	Re-rupture 12.5%; reinfection 13%	4 years (range: 2-8)
Kyriakidis et al., 2023 [14]	Prospective case series; single center	Satisfactory outcomes of patellar tendon reconstruction using Achilles' tendon allograft with bone block after infected total knee arthroplasty	10 knees; all TKA, EMD, PJI	ATA	Allograft failure 20%	34 months (range: 24-53)
Osinga et al., 2020 [17]	Retrospective case series; single center	Orthoplastics in periprosthetic joint infection of the knee: treatment concept for composite soft-tissue defect with extensor apparatus deficiency	160 PJI knees; EMD in 6 (4%)	Free ALT flap with fascia lata; free lateral arm flap with triceps tendon; pedicled MSAP gastrocnemius flap	2/5 reconstructed patients ultimately required arthrodesis for recurrent PJI	3.5 years (range: 0.2-8)
Balato et al., 2023 [3]	Systematic review and meta-analysis; 18 studies	Extensor mechanism reconstruction with allograft following total knee arthroplasty: a systematic review and meta-analysis of Achilles tendon versus extensor mechanism allografts for isolated chronic patellar tendon ruptures	245 knees; PJI 26/245 (11%)	ATA; EMA	Overall failure 23%; EMA 23%; ATA 24%; no significant difference	1.5-5.9 years
Shau et al., 2018 [15]	Systematic review and meta-analysis; 14 studies	Synthetic mesh vs. allograft extensor mechanism reconstruction in total knee arthroplasty - a systematic review of the literature and meta-analysis	204 knees; PJI not specified	ATA; EMA; synthetic mesh	Allograft 23/24%; mesh 25/26%; no significant difference	4.2 years
Rajgopal et al., 2025 [6]	Systematic review and meta-analysis; 32 studies	Extensor mechanism disruption following primary and revision total knee arthroplasty: a systematic review and meta-analysis	774 knees; PJI not specified	Allograft; synthetic mesh; autograft (semitendinosus, gracilis)	Allograft 32.7%; mesh 31%; autograft 0%	Minimum 6 months
Fortier et al., 2024 [18]	Systematic review; 23 studies	Patellar tendon restoration techniques: a systematic review of outcomes for repair and reconstruction methods	738 patients; TKA 150/738; PJI not specified	ATA; EMA; autograft (semitendinosus, gracilis, TFL); synthetic mesh; vastus lateralis flap	Allograft 23%; autograft 0%; mesh 25%	Not reported
De Franco et al., 2022 [4]	Systematic review and meta-analysis; 12 studies	The active knee extension after extensor mechanism reconstruction using allograft is not influenced by "early mobilization": a systematic review and meta-analysis	129 knees; all TKA, EMD; PJI not specified	ATA; EMA	Allograft 8-10%	3.4 years (SD: 1.2)
Vajapey et al., 2019 [19]	Systematic review; 28 studies	Treatment of extensor tendon disruption after total knee arthroplasty: a systematic review	387 knees; PJI not specified	Allograft; autograft; synthetic mesh; flap	Allograft 19%; mesh 19%; re-rupture 25.33%	Minimum 6 months
Oliva et al., 2021 [16]	Systematic review; 25 studies	Complex ruptures of the quadriceps tendon: a systematic review of surgical procedures and outcomes	102 knees; TKA 73/102; PJI not specified	Synthetic mesh; autograft (hamstring); allograft	Mesh 25-32%; allograft 23-32%; autograft 0%	Not reported
Maffulli et al., 2017 [20]	Systematic review; 19 studies	The management of extensor mechanism disruption after total knee arthroplasty: a systematic review	254 knees; all TKA, EMD; PJI not specified	EMA; ATA; synthetic mesh	EMA 0-38%; ATA 0-37%; mesh not analyzed	Not reported

The two studies dedicated exclusively to PJI-associated EMD, by Perry et al. [13] and Kyriakidis et al. [14], enrolled 16 and 10 patients, respectively. The larger systematic reviews pooled data from multiple case series but included heterogeneous populations that variably encompassed both infected and non-infected TKA. Across studies, the population was predominantly female (60-69%) [15], with mean ages ranging from 57 to 72 years [16]. The third primary study, an orthoplastic case series by Osinga et al. [17], reported 160 PJI knees, of which six involved extensor mechanism deficiency, with a median of eight prior operations before orthoplastic reconstruction. Together, these figures underscore the rarity and advanced surgical complexity of PJI-associated EMD as a combined clinical entity.

Risk of Bias

The results of the risk-of-bias appraisal are summarized in Table 2.

**Table 2:** Methodological quality and risk-of-bias appraisal of the included studies JBI: Joanna Briggs Institute critical appraisal checklist; ROBIS: Risk of Bias in Systematic Reviews

Study	Design	Appraisal tool	Overall risk of bias/quality
Perry et al., 2018 [13]	Case series	JBI	Low risk
Kyriakidis et al., 2023 [14]	Case series	JBI	Low risk
Osinga et al., 2020 [17]	Case series	JBI	Low risk
Balato et al., 2023 [3]	Systematic review/meta-analysis	ROBIS	Unclear
Shau et al., 2018 [15]	Systematic review/meta-analysis	ROBIS	Low risk
Rajgopal et al., 2025 [6]	Systematic review/meta-analysis	ROBIS	Low risk
De Franco et al., 2022 [4]	Systematic review/meta-analysis	ROBIS	Low risk
Fortier et al., 2024 [18]	Systematic review	ROBIS	Unclear
Vajapey et al., 2019 [19]	Systematic review	ROBIS	Low risk
Oliva et al., 2021 [16]	Systematic review	ROBIS	Unclear
Maffulli et al., 2017 [20]	Systematic review	ROBIS	Unclear

For reviews rated as unclear, the principal methodological concerns were as follows: in Balato et al. [3], independent dual-reviewer data extraction could not be confirmed and publication bias was not assessed or discussed; in Fortier et al. [18], independent data extraction was not clearly described, the synthesis relied on a general level-of-evidence classification rather than a dedicated risk-of-bias assessment, and publication bias was not examined; in Oliva et al. [16], independent screening was reported but independence of data extraction was not clearly described, quality was graded using the Oxford Centre for Evidence-Based Medicine (CEBM) levels of evidence rather than a dedicated risk-of-bias tool, and publication bias was not addressed; and in Maffulli et al. [20], a dedicated risk-of-bias or quality appraisal tool was not clearly described and publication bias was not assessed.

Types of EMD

The disruptions studied comprised patellar tendon rupture, quadriceps tendon rupture, and patellar fracture [6]; patellar tendon rupture was the most frequently addressed lesion [14]. Most cases were chronic rather than acute (Balato et al. reported chronic disruption in 74% of knees [3]), although chronicity was defined inconsistently: Fortier et al. used a threshold of more than six weeks from injury [18], Oliva et al. used three weeks [16], and Vajapey et al. classified disruptions occurring within three months of TKA as early [19]. Rajgopal et al. distinguished early (within four weeks) from late (after four weeks) failure [6].

Reported mechanisms included impingement and devascularization of the patellar tendon by TKA components [3], eccentric loading on a taut tendon [18], sudden eccentric contraction [16], and postoperative factors such as malrotation, chronic patellar maltracking, compromised local blood supply, infection-related soft-tissue necrosis, and excessive lateral retinacular release [6].

Reconstruction Techniques

Two principal strategies dominate the literature: allograft-based and synthetic mesh-based reconstruction.

Allograft reconstruction employs either an Achilles tendon allograft (ATA) with an attached calcaneal bone block or a whole extensor mechanism allograft (EMA) [3]. Kyriakidis et al. fixed a fresh-frozen ATA by inserting its calcaneal bone block into a rectangular anterior tibial bone window secured with screws and cerclage wires, suturing the tendon to the native extensor mechanism with nonabsorbable sutures [14]. The choice between ATA and EMA is generally guided by the integrity of the native patella: ATA is preferred when the patella is intact, whereas EMA is selected when the patella is deficient or cannot be mobilized [3].

Synthetic mesh reconstruction most commonly employs knitted monofilament polypropylene (Marlex) mesh [13]. Perry et al. anchored the mesh into the tibial medullary canal with bone cement and secured it at the femoral component, closing host tissue over the mesh in a "pants-over-vest" configuration as part of a two-stage exchange arthroplasty [13]. Synthetic mesh has been used more frequently in conjunction with concomitant component revision [15]. Additional mesh types reported include MUTARS mesh [6].

Orthoplastic approaches, described by Osinga et al. [17], included free anterolateral thigh flaps with fascia lata, free sensate extended lateral arm flaps with triceps tendon, and pedicled medial sural artery perforator gastrocnemius flaps for composite soft-tissue defects with extensor apparatus deficiency in PJI. Reported augmentation strategies included cerclage wires, sutures, and transosseous tunnels [18].

Postoperative immobilization in full extension was universally recommended; its duration ranged from four weeks in the early-mobilization arm to eight weeks in the late-mobilization arm of the meta-analysis by De Franco et al. [4] and up to three months in the protocol recommended by Rajgopal et al. [6].

Reconstruction Failure Rates

Direct PJI-specific primary evidence was limited. Perry et al. reported two-year survivorship free of mesh failure of 86%, survivorship free of PJI of 87%, and survivorship free of either PJI or mesh failure of 75% [13]. Kyriakidis et al. reported two treatment failures among 10 patients (20%) [14], while in Osinga et al., two of five reconstructed patients ultimately required arthrodesis because of recurrent infection [17]. In the broader secondary literature, source-reported failure estimates for allograft and synthetic mesh generally fell within the 23-33% range [3,6,15]. Shau et al. reported no statistically significant difference between allograft and synthetic mesh reconstruction [15], while Rajgopal et al. reported failure rates of 32.7% and 31%, respectively [6]. Balato et al. reported similar failure rates for EMA and ATA [3]. These secondary estimates were not pooled or treated as PJI-specific estimates in the present review.

Functional Outcomes

Functional improvement measured by the KSS was consistently documented, although absolute postoperative values varied widely. Perry et al. reported a mean postoperative KSS of 74 after synthetic mesh reconstruction [13]. Shau et al. reported divergent postoperative values for allograft (90.3) and synthetic mesh (53.7) recipients [15]; this difference did not reach statistical significance and probably reflects differences in baseline pathology and indication. Kyriakidis et al. documented improvement of the KSS clinical score from 22.6 to 73.4 and of the functional score from 10 to 55 after ATA reconstruction [14].

Extensor lag improved markedly in both cohorts: from 31° to 3° with Marlex mesh in the series by Perry et al. [13] and from 28.3° to 9° with ATA in that of Kyriakidis et al. [14]. Across pooled analyses, the mean postoperative extensor lag generally fell below 10° [3], although persistent extensor lag of 20° or greater remained the most frequently reported complication [3].

Postoperative range of motion was generally well preserved, with most studies reporting mean flexion between 95° and 110° [13]; Balato et al. noted that 11 of 14 included papers reported mean flexion exceeding 100° [3].

Walking-aid requirement differed by reconstruction type. Balato et al. found that 55% of ATA recipients and 34.5% of EMA recipients required walking aids at the final follow-up [3], whereas Rajgopal et al. reported requirements of 48.6%, 42%, and 40% for synthetic mesh, allograft, and autograft, respectively [6].

Beyond the KSS, patient-reported outcome measures (PROMs) were reported inconsistently across the included evidence. Kyriakidis et al. recorded a Visual Analogue Scale for pain [14], and patient-reported outcome scores were among the measures captured within the broader literature reviewed by Fortier et al. [18], but no dedicated, validated PROM specific to extensor mechanism function was applied consistently across studies, and the version and mode of administration of the KSS itself were not uniformly specified. Standardized PROM reporting therefore remains an evidence gap in this population.

Complications

Persistent or recurrent infection was the most consequential complication, particularly in dedicated PJI cohorts. Perry et al. reported a 13% reinfection rate at two years after Marlex mesh reconstruction within a two-stage exchange [13]. In the broader mixed-cohort review by Shau et al., the pooled postoperative infection rate was 13% [15]. Balato et al. found an overall postoperative infection rate of 7% across allograft reconstructions [3], whereas Vajapey et al. reported a considerably higher rate of 22.67% [19], likely reflecting higher-risk cohorts. In the series by Osinga et al., two of five reconstructed patients ultimately required arthrodesis for recurrent PJI despite initially successful orthoplastic reconstruction [17], illustrating the particularly poor prognosis when PJI and extensor apparatus deficiency coexist.

Re-rupture and graft failure constituted the second most frequent complication category. Vajapey et al. found a re-rupture rate of 25.33% across pooled studies [19], and Rajgopal et al. reported an overall failure rate of 27.5% in the reconstruction group [6]. De Franco et al. found that the timing of postoperative mobilization (four vs. eight weeks) did not materially influence the risk of reconstruction failure or re-rupture [4].

Repair Versus Reconstruction

The clinical advantage of reconstruction over direct repair is supported by Vajapey et al., who reported complication rates of 63.16% for patellar tendon repair and 25.37% for quadriceps tendon repair, compared with 18.8% and 19.2%, respectively, for reconstruction [19]; the authors recommended reconstruction over repair for patellar tendon disruption in both early and late presentations [19]. In the post-TKA setting, Fortier et al. found that allograft reconstruction yielded superior KSS values (79-88) compared with autograft reconstruction (70) [18].

Rehabilitation Timing

De Franco et al. examined whether early (four-week) versus late (eight-week) mobilization after allograft reconstruction influenced outcomes [4]. Failure rates were 13.1% (11/84) in the early-mobilization group and 10.5% (4/38) in the late-mobilization group (p=0.69), with postoperative extensor lag of 9.1° versus 6.5° and mean flexion of 107.6° versus 104.8°, respectively [4]. The source-reported non-significant comparison should not be interpreted as evidence of equivalence or absence of a clinically relevant difference between rehabilitation strategies.

Discussion

Across the broader secondary literature, reported failure estimates for allograft and synthetic mesh reconstruction generally fell within the 23-33% range [3,6,15]. These estimates derive largely from mixed infected and non-infected TKA populations and do not establish statistical equivalence or superiority of either technique. Graft selection should therefore be guided by tissue quality, implant status, the need for concomitant component revision, and institutional experience rather than by an assumed difference in durability [3,13-15].

Consistent with this uncertainty, Baker et al. [7], a single-center retrospective cohort study of 123 extensor mechanism reconstructions, rather than a meta-analysis, reported a substantial risk of infection, revision, and extensor lag across both allograft and synthetic mesh reconstructions, underscoring that technique-specific risk estimates from single-center cohorts should be interpreted cautiously alongside the broader pooled literature.

The divergence in postoperative KSS between allograft (90.3) and synthetic mesh (53.7) recipients reported by Shau et al. [15] most likely reflects selection bias rather than a true technique effect: synthetic mesh was used more frequently alongside concomitant component revision [15], and the mean interval from diagnosis to treatment was substantially shorter in the mesh group (three months) than in the allograft group (19.5 months) [15], suggesting that mesh was preferentially chosen for more acute and surgically complex presentations. The dedicated PJI cohort of Perry et al. achieved a postoperative KSS of 74 with synthetic mesh [13], which lies within the clinically functional range and may more accurately represent expected outcomes for mesh reconstruction in the infected setting.

The differential walking-aid requirement, 55% for ATA, 34.5% for EMA, and 48.6% for synthetic mesh [3,6], may reflect differences in graft biology and fixation mechanics rather than inherent inferiority of any one technique. EMA provides a composite graft with patellar bone stock that may enable more effective quadriceps force transmission, whereas ATA and synthetic mesh rely on soft-tissue-to-soft-tissue healing or cement fixation, respectively; in the biologically compromised field characteristic of PJI, this distinction may carry particular relevance.

Reconstruction is clearly preferable to direct repair, particularly for patellar tendon disruption. Complication rates of 63.16% for patellar tendon repair versus 18.8% for reconstruction [19] underscore the limitations of primary repair in the altered biomechanical and biological environment of the post-arthroplasty knee. For quadriceps tendon disruption, decision-making may depend more on residual stump quality and local tissue condition, with selected primary repair potentially remaining feasible in favorable circumstances [16,19]. For post-TKA quadriceps tendon rupture specifically, synthetic mesh was the most frequently selected technique (53% of cases reported by Oliva et al. [16]), with a favorable mean postoperative extensor lag of 6.3° in this subgroup.

Infection control remains central to long-term outcome. Perry et al. reported two-year survivorship free of PJI of 87% and survivorship free of both PJI and mesh failure of 75% [13], while Osinga et al. showed that recurrent infection may ultimately necessitate arthrodesis [17]. PJI-associated EMD therefore represents a compounded reconstructive problem in which infection eradication is at least as important as restoration of extensor continuity.

Findings converge and diverge in informative ways when the two PJI-dedicated primary studies are considered separately from the broader mixed-cohort reviews: Perry et al. [13] and Kyriakidis et al. [14] report PJI-specific reconstruction outcomes directly, whereas the wider reviews additionally contribute functional and complication data drawn from mixed infected and non-infected populations. This comparison is descriptive rather than a formal sensitivity analysis. Crude failure proportions are also time-dependent and are not directly comparable across studies with materially different follow-up durations, and included sources vary in whether they report outcomes per patient, per knee, or per reconstruction; these units are not treated as interchangeable in this synthesis.

Despite this challenging environment, functional improvement is achievable: most studies reported postoperative extensor lag below 10°, mean flexion between 95° and 110°, and KSS values in the functional range [3,13,14]. Persistent extensor lag of 20° or greater nonetheless remained the most common complication [3,19], reflecting the difficulty of reliably reproducing native extensor mechanism mechanics in a scarred and biologically compromised field.

From a practical standpoint, synthetic mesh offers lower cost, near-universal availability, absence of disease transmission risk, and potentially reduced graft elongation over time [15]. Allograft provides a more anatomically faithful biological reconstruction but requires tissue-banking infrastructure and carries a theoretical disease transmission risk. In the infected setting, the choice between these approaches may be best guided by whether a single-stage or two-stage exchange is planned: Perry et al. demonstrated successful incorporation of mesh into a two-stage protocol [13], whereas Kyriakidis et al. showed that ATA performed after confirmed infection eradication achieved 80% success [14].

Primary data on autograft reconstruction specifically in the PJI-associated setting remain very limited within the identified evidence, and this is noted here as a distinct gap rather than assumed to be equivalent to the allograft- and mesh-specific findings discussed above.

Limitations

This review has several limitations. First, the evidence base consists predominantly of small retrospective case series and of systematic reviews pooling heterogeneous cohorts, which limits the strength of any conclusion. Second, most included systematic reviews combine infected and non-infected TKA populations, making it difficult to isolate outcomes attributable specifically to the PJI setting. Third, considerable variation in the definitions of reconstruction failure, disruption chronicity, and rehabilitation protocols impedes direct comparison. Fourth, the included evidence is predominantly of low level (Level IV observational studies and secondary syntheses), which limits certainty in the pooled estimates even after formal quality appraisal. Fifth, because several of the included systematic reviews draw on overlapping primary case series, individual patients may be represented in more than one source; the core synthesis was therefore based on the three primary studies and the reviews treated as supporting context, but residual double-counting within the pooled estimates cannot be fully excluded. No formal quantitative overlap analysis such as the corrected covered area was performed; to avoid treating overlapping reviews as independent evidence, review-level pooled estimates were not combined into a new summary estimate, and the synthesis was anchored on the primary studies directly addressing PJI-associated EMD, with the broader systematic reviews used as contextual secondary evidence. Sixth, publication bias may favor studies reporting more favorable outcomes. Seventh, study screening and selection were performed primarily by a single reviewer, with inclusion confirmed by consensus with a second author rather than through a fully independent dual-reviewer process, which may increase the risk of selection error. Eighth, included sources varied in whether outcomes were reported per patient, per knee, or per reconstruction, and these units were not treated as interchangeable. Finally, the review was not prospectively registered in PROSPERO, which reduces protocol transparency; prospective registration is recommended for any future update.

Future Directions

Prospective multicenter studies that specifically enroll patients with PJI-associated EMD, using standardized definitions of infection eradication, disruption chronicity, and reconstruction failure, are needed to generate higher-level evidence. Head-to-head comparisons of allograft and synthetic mesh in the infected setting, together with PROMs beyond the KSS and long-term functional data, would meaningfully advance evidence-based decision-making in this challenging population. A targeted check of major arthroplasty registries (e.g., the Australian Orthopaedic Association National Joint Replacement Registry or the Swedish Knee Arthroplasty Register) did not identify registry-based data reporting PJI-associated extensor mechanism reconstruction technique and outcome with sufficient granularity for the present question; improved registry capture of reconstruction technique and infection status would be a valuable direction for future data collection, alongside standardized definitions of infection status, reconstruction technique, and reconstruction failure, and the use of validated PROMs.

## Conclusions

Extensor mechanism reconstruction in the setting of PJI after TKA is a technically demanding limb-salvage procedure with a guarded functional prognosis. The available evidence indicates a broadly similar range of reported failure rates between allograft and synthetic mesh techniques (consistently between 23% and 33% across the identified evidence, though drawn largely from broader secondary reviews rather than a pooled PJI-specific estimate, and without a formal statistical comparison between techniques). Durable success depends chiefly on effective infection eradication, adequate soft-tissue coverage, and individualized operative planning matched to the defect, rather than on graft material alone. Given the complexity and compounded morbidity of this combined pathology, management at the interface of orthopedic and reconstructive surgery within a multidisciplinary framework is recommended. Future prospective studies with standardized outcome reporting and dedicated PJI cohorts are required to establish definitive, evidence-based guidance.
